# Tibial Spine Avulsion Fracture in an Adult: An Uncommon Occurrence With Surgical Implications

**DOI:** 10.7759/cureus.83261

**Published:** 2025-04-30

**Authors:** Felix Rivera Troia, Carlos J Perez Lopez

**Affiliations:** 1 Orthopaedic Surgery, Ponce Health Sciences University, Ponce, PRI

**Keywords:** acl, anterior tibial spine, arthroscopy and sports related injuries, avulsion fracture, trauma and sports injury

## Abstract

Tibial spine avulsion fractures (TSAFs) are an uncommon cause of knee pain in children and adolescents and are exceedingly rare in adults. In skeletally mature individuals, these injuries typically result from high-energy trauma, such as motor vehicle accidents, and are often associated with concomitant knee injuries. We present the case of an adult female who sustained a TSAF after a fall while skateboarding. Given the nature of the injury, the risks and benefits of nonoperative versus operative management were discussed, and the patient elected to undergo surgery. Arthroscopic fixation of the tibial spine via the suture pull technique, along with a concomitant repair of the posterior horn of the medial meniscus, was performed and yielded satisfactory results at the final follow-up visit. This case highlights an uncommon injury in adults while demonstrating the effectiveness of the arthroscopic suture pull fixation in achieving favorable clinical outcomes.

## Introduction

Tibial spine avulsion fractures (TSAFs) are uncommon injuries that primarily occur around the ages of eight to 14 and account for less than 6% of all pediatric knee injuries [1]. In skeletally immature individuals, the anterior cruciate ligament (ACL) is stronger than the incompletely ossified tibial spine, making it prone to injury during common abrupt movements such as pivot-shift rotation, knee hyperextension, or direct trauma [2]. Meyers and McKeever defined a classification system for this type of injury to help guide appropriate management [3]. For type I and II fractures, which involve minimal displacement of the anterior margin or displacement of two-thirds with an intact posterior hinge, respectively, conservative management through immobilization is sufficient for adequate bone union. For type III fractures that involve complete dislodgement of the bony fragment, surgical intervention is often employed [3,4].

Although rare in children, TSAFs are even more uncommon in adults and typically result from high-velocity motor vehicle accidents [5]. Furthermore, studies suggest that in skeletally mature individuals, these fractures are frequently associated with concomitant knee injuries compared to the pediatric population [6]. Few reports in the literature have documented this type of injury in adults [5,7-12], with most cases resulting from high-energy trauma. Here, we present the case of an adult female patient who sustained a fall while skateboarding and was diagnosed with a TSAF. It also highlights the efficacy of the surgical technique used in achieving satisfactory clinical outcomes.

## Case presentation

**Figure 1:**
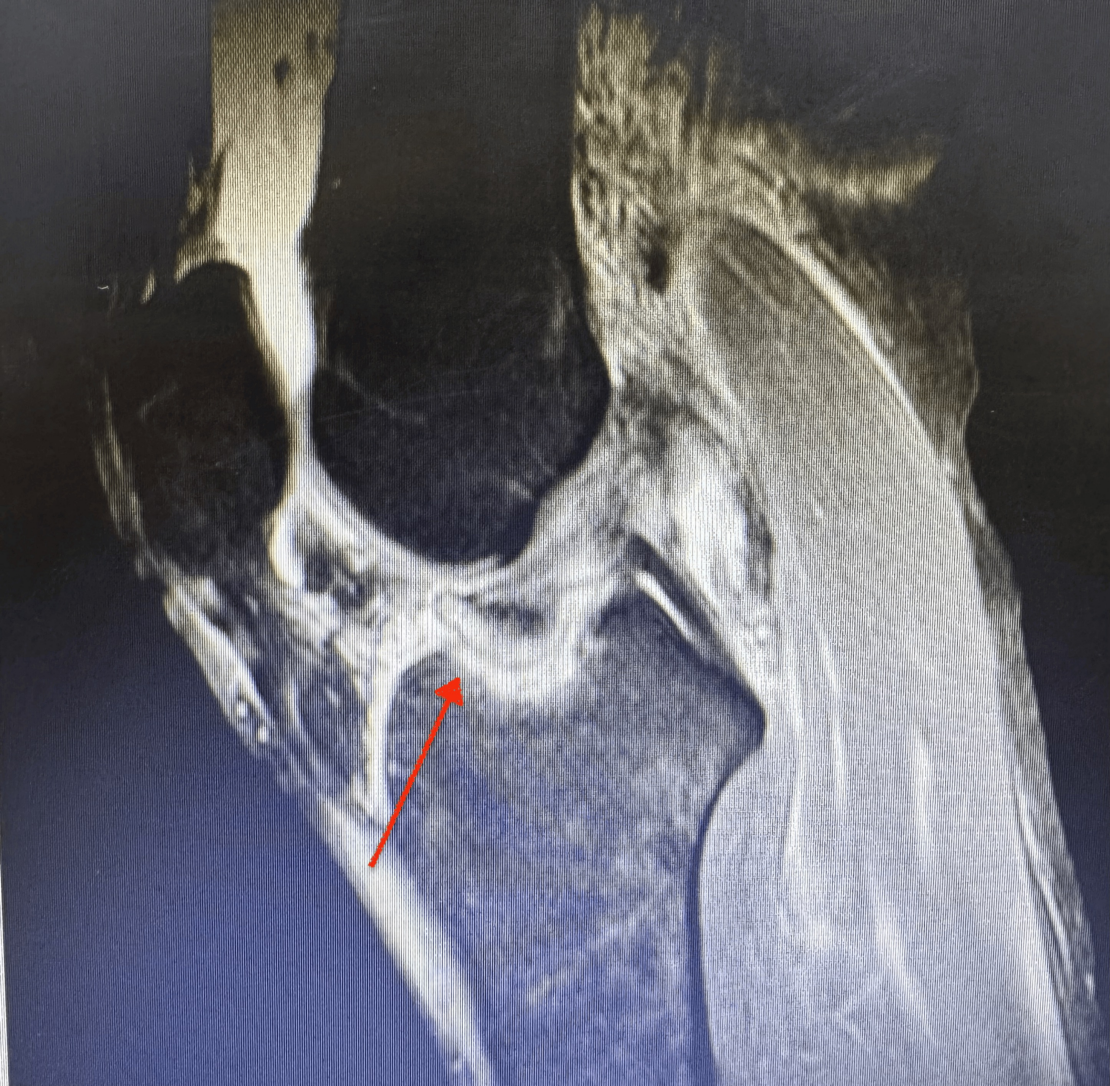
MRI sagittal view of the left knee demonstrating the site of the tibial spine avulsion fracture (red arrow)

This is the case of a 38-year-old female competitive downhill skateboarder, with no relevant past medical history and who was referred to the orthopedic clinic. She had sustained a knee injury while practicing her sport, one week prior to the visit. The patient reported dismounting her skateboard at high speed while going downhill, stepping on her left foot, and feeling a sprain on her left knee. The pain was described as severe and was rated as 9/10 on the pain scale throughout her knee. But it was more pronounced in the medial joint line. An initial evaluation by an adult reconstruction specialist involved a magnetic resonance image (MRI) of the patient's left knee revealed an avulsion fracture of the tibial spine (Figure 1). 

Given this finding and the nature of the injury, the patient was referred to the sports medicine clinic for further evaluation. On observation, the patient was unable to bear weight on the left lower extremity and required bilateral crutches for ambulation. Physical examination revealed moderate swelling around the left knee and diffuse joint line tenderness on palpation. Lachman exam showed laxity, but this was unreliable due to the patient's apprehension in this acute state. Her range of motion (ROM) was significantly limited, though no crepitus was noted and the extremity remained neurovascularly intact. 

During the initial visit, arthrocentesis of the left knee was performed, yielding 30 cc of bloody fluid, which provided some pain relief. Furthermore, a discussion was held regarding the potential risks and benefits of conservative management versus surgical intervention, and the patient opted for a surgery. Subsequently, she was scheduled for an arthroscopic fixation of the TSAF of the left knee via the suture pull technique the next week.

Surgical technique

After informed consent was obtained, the patient was taken to the operating room where she was placed in the supine position with all prominences well-padded, and with good head and cervical alignment. The knee was examined under general anesthesia and the site was prepped and draped in the usual sterile manner. 

**Figure 2:**
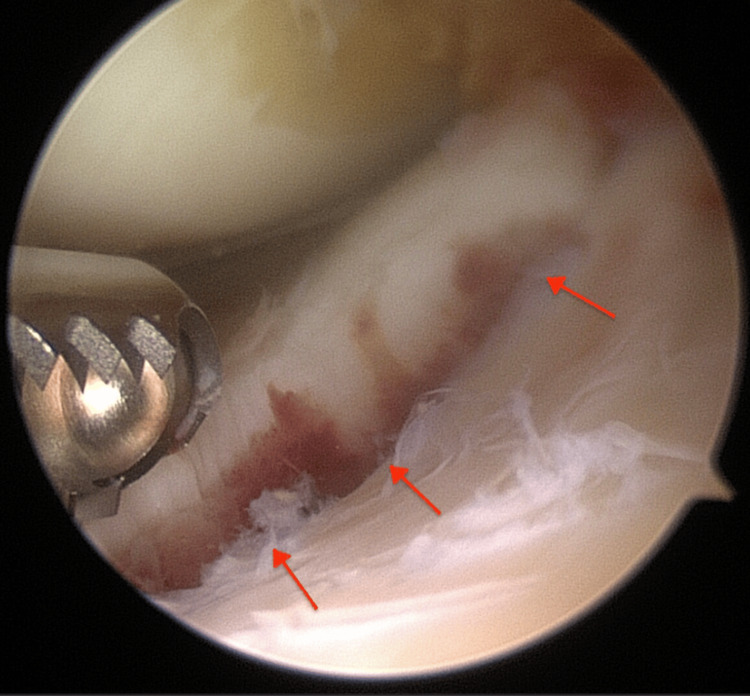
The avulsed bony fragment (red arrows)

**Figure 3:**
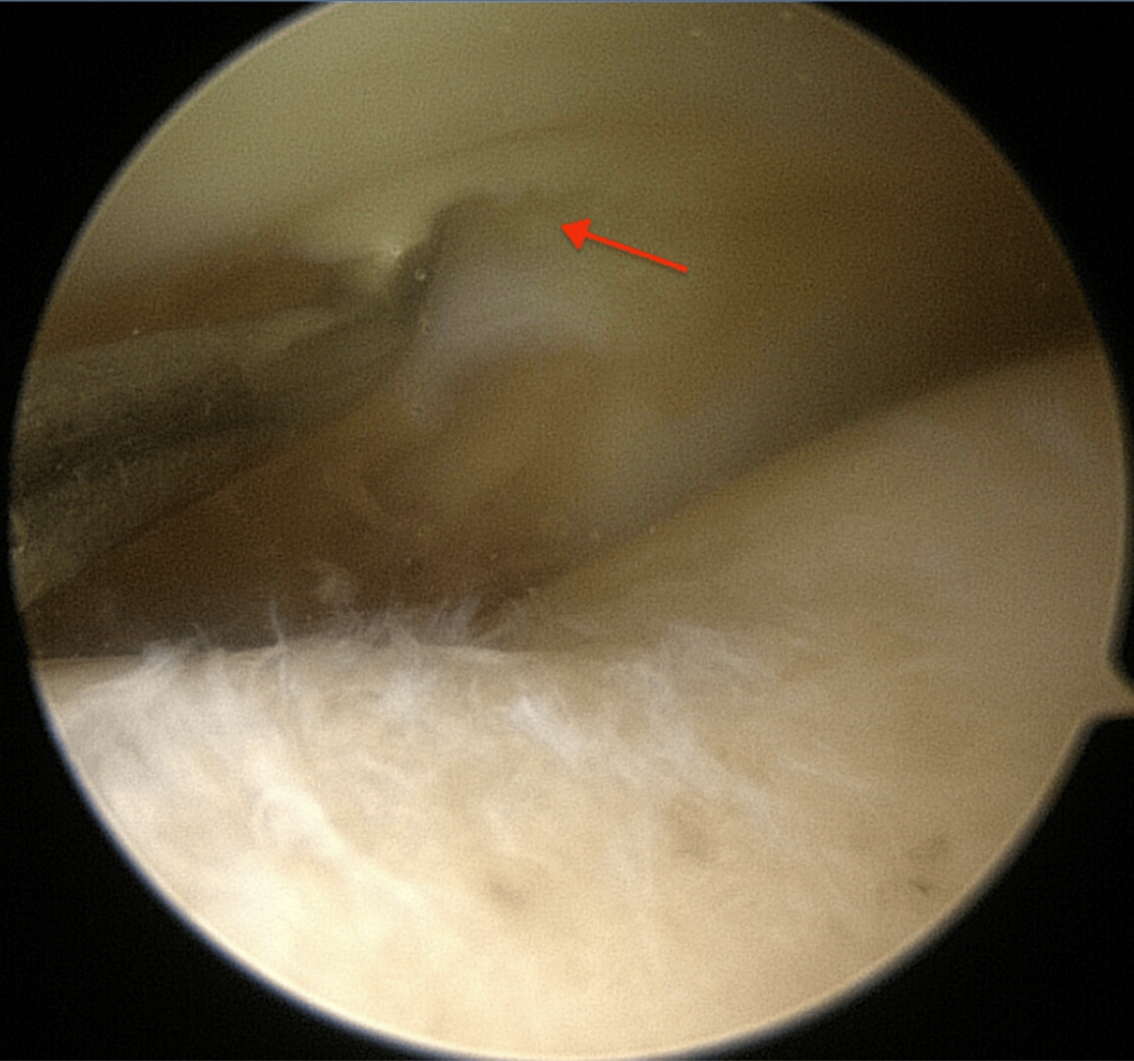
Probe demonstrating horizontal tear (red arrow) underneath the posterior horn of the medial meniscus

Anterolateral and anteromedial portals were established and a diagnostic arthroscopy was performed which revealed a TSAF along with a horizontal tear on the undersurface of the posterior horn of the medial meniscus (Figures 2, 3).

**Figure 4:**
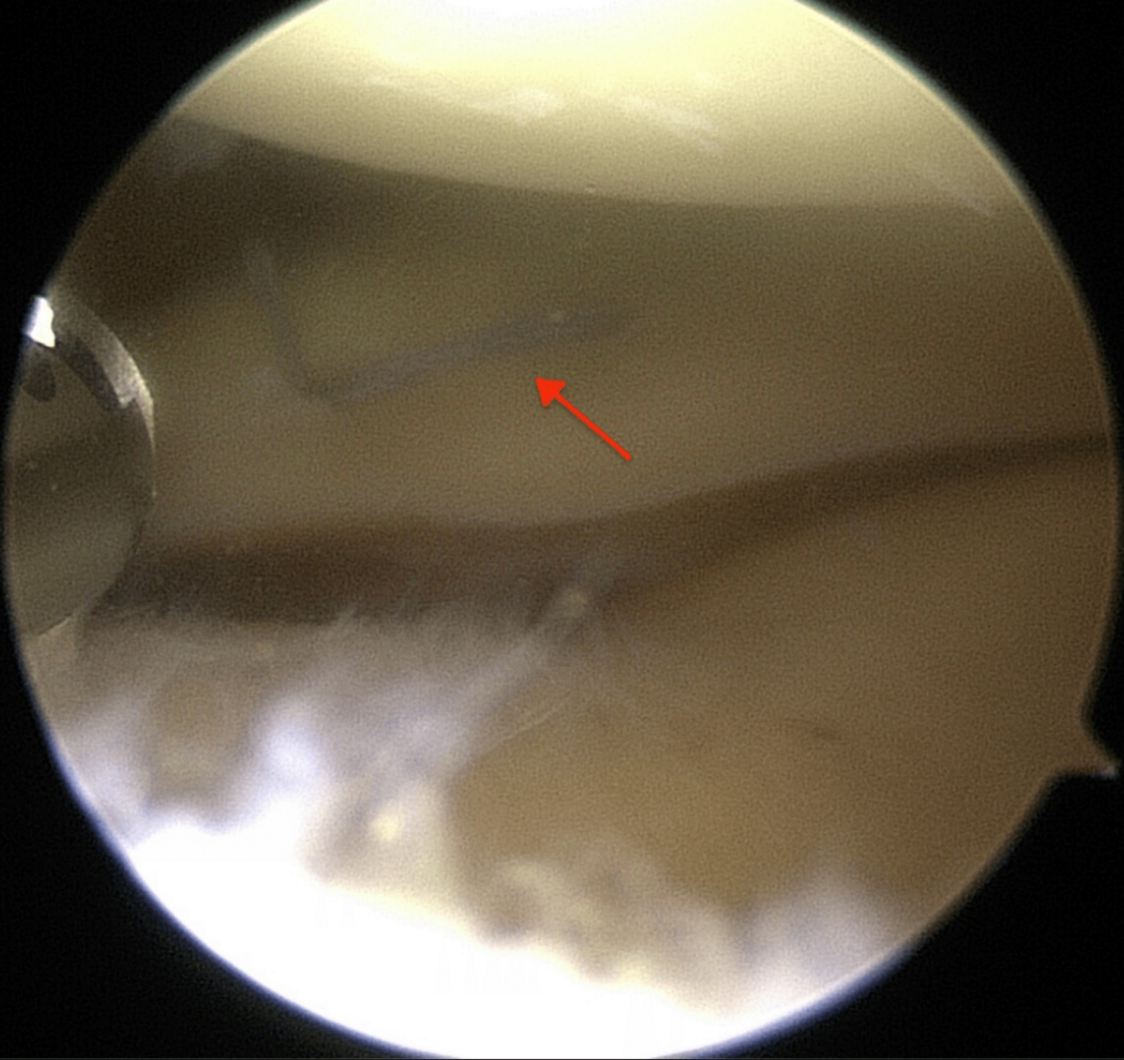
The medial meniscus tear fixed with an all inside technique (red arrow)

The medial meniscus was probed and deemed amenable to repair by means of an all inside meniscal repair device (DePuy Mitek, Inc., Massachusetts, USA; Figure 4).

**Figure 5:**
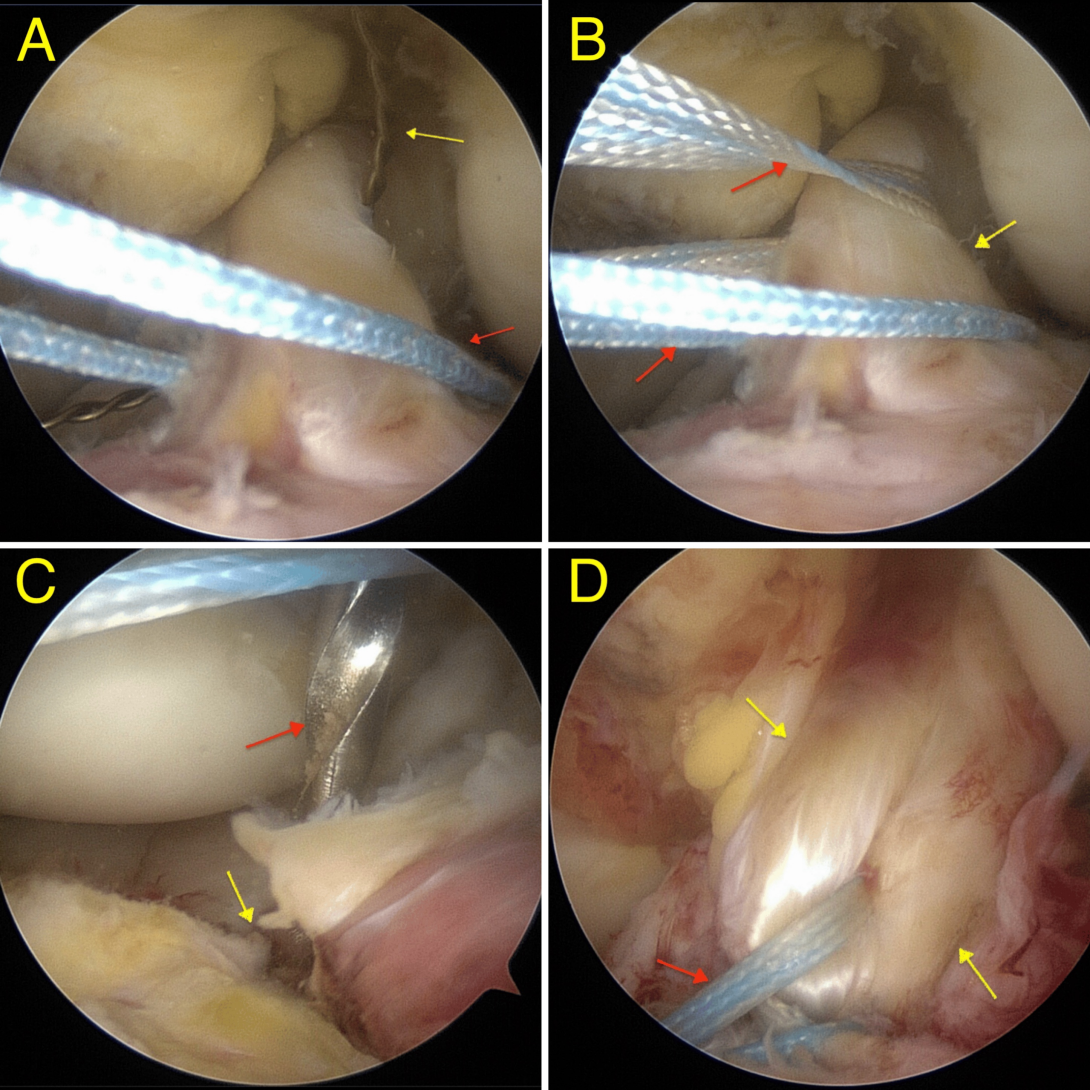
The suture-pull technique A: First lower suture passed (red arrow) and superior suture passer in place (yellow arrow) for shuttling the second suture. B: High-strength sutures (red arrows) passed through the anterior cruciate ligament (ACL; yellow arrow) in preparation for fixation to the tibial spine. C: Drill tip (red arrow) passing through one of the tibial tunnels (yellow arrow) created prior to final fixation. D: Final fixation of the bony fragment (reduced and not visible on image) via the pull-through technique using high-strength sutures (red arrow) through the ACL (yellow arrows).

The intercondylar notch was then addressed to assess the avulsion site via the suture pull technique. Two high strength suture tapes (Arthrex, Florida, USA) were passed through the ACL for fixation of the tibial spine fracture. An anteromedial incision of the proximal tibia was performed with dissection carried down to bone. Two bone tunnels approximately 15 mm apart were drilled using the ACL tibial aimer (3 mm wide; DePuy Mitek, Inc., Massachusetts, USA). The sutures were subsequently passed through the bone tunnels with the knee in the extended position and suture fixation was performed in the tibia while performing a posterior drawer maneuver and pulling the sutures. A very good fixation was obtained (Figures 5A-5D).

The excess sutures were removed and the joint was copiously irrigated and drained. Finally, the incision over the tibia was irrigated and closed in layers.

Following the surgery, the patient was discharged with pain medication, a knee brace, and was instructed to not place any weight on the affected extremity while using bilateral crutches for ambulation. At her first postoperative visit, the sutures were removed, and the patient reported to be managing her pain well. She was referred to physical therapy to aid in her recovery. After four weeks, the patient was gradually encouraged to bear as much weight as tolerated on the affected extremity. She was then prescribed a standard ACL outpatient rehabilitation program. By the final one year follow-up, she reported full return to competitive downhill skateboarding with full ROM, no effusions, very good extremity strength, and balance and control.

## Discussion

TSAFs are a rare cause of knee injuries in the pediatric population and affect three out of every 100,000 patients per year [13]. Characterized by a bony avulsion of the tibial spine at the ACL insertion site, these injuries result from a strength imbalance between the ligament and the incompletely ossified bone and commonly occur in young athletes during organized sports [14]. In contrast, in adults, they are typically the result of high-energy trauma, such as motor vehicle accidents [5].

In skeletally mature individuals, TSAFs are often accompanied by concomitant meniscal tears [9], a notable consideration, as entrapped soft tissue between the avulsed fragment and its bed can impede proper reduction, often necessitating surgical intervention [15]. In our case, a diagnostic arthroscopy revealed a tear amenable to repair in the posterior horn of the medial meniscus. However, the lesion was not severe enough to cause interposition at the fracture site. 

Various surgical techniques have been described for the fixation of TSAFs [10-12]. Chawda et al. reported excellent clinical and radiographic outcomes in a series of 10 patients who underwent open reduction and internal fixation (ORIF) for TSAFs, advocating for this approach as a less technically demanding and more cost-effective option compared to other techniques [10]. In contrast, Pandey et al. utilized the arthroscopic suture pull-out technique, similar to the method used in our patient, and reported comparable outcomes, with the added benefit of preserving the terminal knee extension [12]. Nonetheless, a study by Shimberg et al. comparing ORIF with arthroscopic fixation found no significant differences among outcomes or complication rates between the two approaches, suggesting that both techniques are viable treatment options [11].

TSAFs require careful consideration due to their potential association with concomitant knee injuries such as meniscal tears. Surgical intervention, particularly using arthroscopic techniques, can offer favorable outcomes, as demonstrated in this case and in others [2,5,8,11-13,15]. Further studies will continue to refine our understanding of these injuries and help establish the best practices for treatment, particularly in this unique population where such injuries are not commonly encountered.

## Conclusions

This case highlights the rare occurrence of a TSAF in an adult female patient. While these fractures are primarily seen in the pediatric population, their presence in skeletally mature individuals often requires surgical intervention, particularly when associated with meniscal tears or other soft tissue injuries. Arthroscopic fixation via the suture pull technique can be considered by experienced surgeons as it may yield good results, as seen in this case. The technique allows for good reduction, preservation of the knee's ROM, and satisfactory clinical outcome. This case contributes to the growing body of literature on TSAFs in adults and reinforces the role of arthroscopic techniques in achieving favorable results.
